# Deciphering the Temporal Transcriptional Dynamics and Key Regulatory Networks of *Pyrus betulifolia* in Response to PEG-Induced Osmotic Stress

**DOI:** 10.3390/biology15060459

**Published:** 2026-03-11

**Authors:** Ziyi Zhang, Ke Li, Wenxuan Chu, Yan Zeng, Yutong Zhu, Ruigang Wu, Qingjiang Wang

**Affiliations:** School of Landscape and Ecological Engineering, Hebei University of Engineering, Handan 056038, China; y2233451088@163.com (Z.Z.); keli505@163.com (K.L.); 88j88@163.com (W.C.); 18854861590@163.com (Y.Z.); 18265025836@163.com (Y.Z.)

**Keywords:** drought stress, *Pyrus betulifolia*, RNA-seq, transcription factors, PEG-induced osmotic stress

## Abstract

Drought stress severely restricts the growth of pear trees. As a widely used drought-tolerant rootstock, *Pyrus betulifolia* exhibits stable growth performance; however, the molecular mechanisms underlying its drought tolerance remain to be elucidated. In this study, we investigated the molecular responses of *P. betulifolia* leaves to osmotic stress induced by 20% PEG-4000 using time-series RNA-seq technology. A total of 3745 differentially expressed genes were identified, with transcriptional changes peaking at 6 h, indicating a critical phase of transcriptional reprogramming during drought response. Genes associated with osmotic adjustment (e.g., P5CS) and oxidative stress responses (e.g., SOD and POD) were significantly upregulated between 6 and 12 h. Weighted gene co-expression network analysis (WGCNA) identified three distinct temporal modules and screened out NF-Y, RVE1, COL9, COL6, C2C2 zinc finger proteins, and Pseudo ARR-B as putative key regulators, whose expression patterns were validated using qRT-PCR. Collectively, these results provide a comprehensive view of the temporal transcriptional dynamics of drought response in *P. betulifolia* and offer valuable candidate gene resources for further functional studies and drought tolerance breeding.

## Simple Summary

Drought is a major environmental constraint limiting crop growth and fruit yield. Due to its high drought tolerance, *Pyrus betulifolia* is widely used as a rootstock for pear cultivation; however, its molecular response mechanisms and transcriptional regulatory features under drought stress remain insufficiently characterized. In this study, we systematically analyzed the temporal transcriptomic responses of pear leaves under PEG-induced osmotic stress conditions. The results revealed a rapid transcriptional reprogramming stage, with the most pronounced gene expression changes occurring at 6 h after stress treatment. During this stage, multiple biological pathways associated with osmotic adjustment, oxidative stress responses, and cellular protection were significantly enriched, suggesting their potential involvement in drought adaptation. Furthermore, co-expression network analysis identified a set of putative key regulatory genes, providing valuable candidates for future functional validation. Collectively, this study delineates the temporal dynamics of osmotic-responsive transcriptional regulation in *P. betulifolia* and identifies key candidate genes, providing a molecular framework for further elucidation of stress tolerance mechanisms and supporting drought resistance breeding efforts.

## 1. Introduction

As one of the predominant abiotic stressors globally, drought severely restricts crop growth and productivity, thereby constituting a persistent challenge to food security and agricultural sustainability [1,2]. Drought stress induces a series of physiological disorders, such as water deficit, disrupted osmotic regulation, and oxidative damage, which markedly suppress crop growth and development and ultimately result in substantial yield loss. Pear (*Pyrus* spp.), a prominent temperate deciduous fruit tree in the Rosaceae family, boasts a long history of cultivation and high economic value [3]. However, pear cultivation frequently faces abiotic stresses such as drought, which significantly inhibit growth and result in reduced fruit yield [4]. Utilizing drought-resistant rootstocks is an effective strategy to improve water use efficiency and environmental adaptability in pear trees. *Pyrus betulifolia*, a wild rootstock resource characterized by a vigorous root system and strong drought tolerance, plays a critical role in drought resistance breeding. Therefore, elucidating its underlying molecular mechanisms of drought resistance is of paramount importance.

Plant response to drought stress is a complex and dynamic process involving multi-gene coordination and the interaction of multiple signaling pathways. Under water deficit, plant cells undergo a series of physiological and biochemical changes, including reduced turgor pressure, oxidative damage induced by the burst of reactive oxygen species (ROS), and osmotic imbalance. To cope with these injuries, plants have evolved sophisticated molecular regulatory mechanisms. By reprogramming their transcriptome and activating ABA-dependent and -independent signaling pathways, plants regulate stomatal closure, induce the synthesis of osmoregulatory substances (e.g., proline and soluble sugars), and enhance antioxidant enzyme activities to re-establish cellular homeostasis [5]. In recent years, transcriptomics has become a powerful tool for systematically revealing global gene expression profiles and mining key regulatory genes under abiotic stress [6]. Through RNA-seq technology, researchers have identified numerous transcription factors (TFs) involved in drought response in various plants. For instance, GbMYB5 positively regulates drought tolerance in cotton [7]; OsbZIP62 participates in the ABA signaling pathway to positively regulate drought tolerance in rice by modulating the expression of stress-related genes [8]; and SlNAC6 influences endogenous ABA levels by regulating the transcription of ABA-related genes, thereby participating in tomato development and drought response [9]. These transcription factors play important roles in critical physiological processes such as stomatal movement, osmolyte synthesis, ROS scavenging, and hormone signaling, constituting the molecular regulatory hubs of plant drought response.

Although research on crop drought resistance is extensive, studies on the molecular mechanisms in pear rootstocks, particularly *P. betulifolia*, remain relatively lagging. Current research primarily focuses on physiological and biochemical measurements (e.g., photosynthetic rate, enzyme activity changes) or the cloning and functional verification of single genes, lacking a systematic analysis of the dynamic transcriptional regulatory network at the whole-genome level. Plant drought resistance is a continuous process that changes dynamically over time—ranging from early signal perception to intermediate defense activation, and finally to late adaptation and recovery. Consequently, focusing on a single time point often fails to capture the full picture. Moreover, traditional Differential Gene Expression (DGE) analysis often struggles to pinpoint core regulatory factors. Weighted Gene Co-expression Network Analysis (WGCNA) [10], as an advanced systems biology method, utilizes gene expression correlations to construct networks. It effectively identifies co-expressed gene modules and core hub genes within them. While WGCNA has been successfully applied to mine regulatory factors for complex traits in crops like maize and soybean, its application in pear rootstock drought research remains limited.

Natural drought is a slow and progressive process involving numerous variable factors that are difficult to control. In contrast, polyethylene glycol (PEG), as a non-absorbable, non-metabolizable, and non-toxic osmotic agent, allows for precise quantification of stress levels, making it more suitable for studying the molecular mechanisms of plant drought response [11]. Therefore, this study employed a time-course experimental design (0, 3, 6, 12, 24, and 48 h) to investigate the responses of tissue-cultured *P. betulifolia* plantlets to PEG-induced osmotic stress. Utilizing high-throughput transcriptome sequencing, we systematically analyzed the dynamic patterns of gene expression in *P. betulifolia* leaves under drought stress. We identified critical stages of the drought response and their characteristic differentially expressed genes. Furthermore, we mined core co-expression modules and hub transcription factors closely related to stress duration. This study provides important candidate gene resources for elucidating the molecular mechanisms of drought resistance in *P. betulifolia* and facilitating drought resistance breeding.

## 2. Materials and Methods

### 2.1. Plant Materials and Growth Conditions

The tissue culture of *Pyrus betulifolia* was subcultured on Murashige and Skoog solid medium supplemented with 0.5 mg L^−1^ benzylaminopurine and 0.1 mg L^−1^ naphthylacetic acid, under a 16/8 h light/dark cycle at 25 °C. Subsequently, 1-month-old *Pyrus betulifolia* tissue culture seedlings were subjected to osmotic stress treatment (20% PEG-4000 Is obtained from Sigma Reagent Co., Ltd., Burlington, MA, USA) in the same subculture medium. Leaf samples were collected at 0 h (control group), 3 h, 6 h, 12 h, 24 h, and 48 h. At each sampling time point, leaves from 10 tissue-cultured seedlings with uniform growth were pooled to form a single biological replicate. Three biological replicates were prepared for each time point, with approximately 2 g of leaf tissue collected per replicate and packaged in aluminum foil bags. The samples were rapidly frozen in liquid nitrogen and stored at −80 °C for RNA extraction and subsequent transcriptome analysis.

### 2.2. RNA-Seq Data Processing, Transcript Assembly, and Expression Analysis

Raw reads were first subjected to quality assessment and adapter detection using the FastQC v0.20.0 [12] package. Subsequently, low-quality reads and adapter-contaminated sequences were removed using NGSQC v2.3.3 [13]. The resulting clean reads were aligned to the *Pyrus betulifolia* reference genome (http://gigadb.org, accessed on 5 October 2025) using HISAT2 v2.1.0 [14], and transcript assembly and quantification were performed with StringTie v2.1.3b [15]. Gene-level count data were normalized using DESeq2 v1.26.0 [16], which was also employed to calculate fold changes and assess differential expression based on a negative binomial distribution model. Differentially expressed genes (DEGs) were identified according to log_2_ fold change values, with genes showing log_2_ fold change ≥ 1 and q-value ≤ 0.05 classified as upregulated and those with log_2_ fold change ≤ −1 and q-value ≤ 0.05 classified as downregulated.

### 2.3. Principal Component Analysis (PCA)

Principal component analysis (PCA) was performed to explore the expression patterns among different samples. Raw count data were first transformed using the DESeq2 v1.26.0 package, after which PCA was conducted with the princomp function in R V4.4.1. The resulting components were visualized using the factoextra 1.0.6 and ggplot2 packages V3.5.0.

### 2.4. Gene Ontology (GO) and KEGG Pathway Enrichment Analysis

Gene Ontology (GO) annotation was performed using the Blast2GO v2.10.1 software, with a significance threshold of *p* < 0.05. Directed acyclic graphs and hierarchical tree maps were generated using Blast2GO v2.10.1 and ReviGO (http://revigo.irb.hr/, accessed on 1 July 2025). KEGG pathway enrichment analysis and gene-set annotation were conducted with KOBAS 2.0. Query gene sequence files were first retrieved from the Rosaceae Genome Database (https://www.rosaceae.org, accessed on 1 October 2025) and aligned, and the resulting files were used as input for KOBAS. Functional annotation and pathway enrichment were subsequently carried out based on the Kyoto Encyclopedia of Genes and Genomes (KEGG) database. All heatmaps were generated using the heatmap function implemented in TBtools-II V2.435.

### 2.5. Identification of Gene Co-Expression Modules

Weighted gene co-expression network analysis (WGCNA) was performed in R to identify highly correlated gene modules based on the RNA-seq expression matrix. Gene expression and variation were first filtered using the DCGL package V2.1.2. The adjacency matrix was then transformed into a topological overlap matrix (TOM) using TOM-based similarity algorithms, and modules with highly correlated eigengenes (correlation coefficient > 0.8) were merged. Network visualization was conducted using Cytoscape v3.0.0.

### 2.6. Quantitative Expression Analysis by RT-qPCR

Gene-specific primers for pear were designed using Primer-BLAST (https://www.ncbi.nlm.nih.gov/tools/primer-blast, accessed on 1 October 2025), with sequences listed in Table S1. Quantitative real-time PCR (qPCR) was performed using cDNA templates from pear samples collected at different time points to analyze pear gene expression under drought stress. Each 20 µL reaction contained 10 µL of 2× Taq Pro Universal SYBR qPCR Master Mix (Vazyme Biotech Co., Ltd., Nanjing, China), 0.5 µL each of forward and reverse primers, 1 µL cDNA, and 8 µL ddH_2_O. The thermal profile included initial denaturation at 95 °C for 30 s, followed by 40 cycles of 95 °C for 5 s and 55 °C for 34 s. Relative expression levels were calculated via the 2−ΔΔCT method with three biological and technical replicates. Statistical analyses were performed using SPSS 22.0 software, while data visualization was generated using Excel 2013, TBtools-IIv2.326, and Graphpad Prism 10.1.2.

## 3. Results

### 3.1. Transcriptomic Analysis Based on RNA-Seq

**Figure 1:**
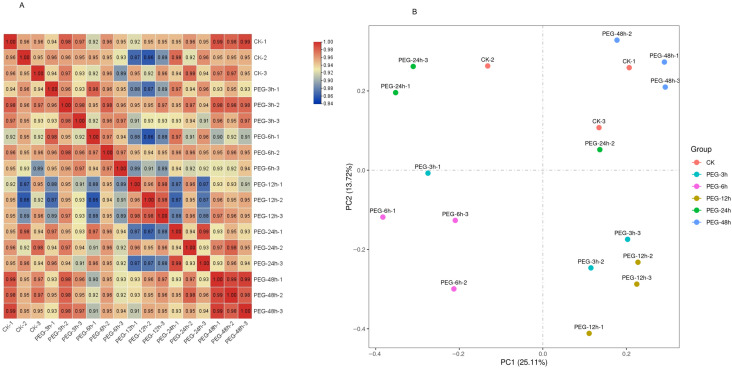
Transcriptome analysis of Pyrus betulifolia under PEG-induced osmotic stress. (**A**) Correlation among the three biological replicates within each treatment group, based on FPKM expression values. (**B**) Principal component analysis (PCA) of the transcriptome data, with three biological replicates for each sample.

To investigate the transcriptional response dynamics of *Pyrus betulifolia* to drought stress, we collected leaf samples from in vitro cultured *Pyrus betulifolia* tissue culture seedlings under 20% PEG-4000 stress treatment and performed RNA sequencing at different time points (0–48 h). Overall, after removing low-quality reads and adapters, a total of 131.01 Gb of data was obtained, with each sample’s Clean Data exceeding 6.31 Gb. Among these, 91.63% of the sequences were successfully aligned to the *Pyrus betulifolia* genome (Table S2). To assess the reliability of the experimental data, an inter-sample correlation analysis was performed. The results indicated that the correlation coefficients between the three biological replicates under the same treatment conditions were all greater than 0.9 (Figure 1A), demonstrating the high reproducibility of the experimental samples (Table S3). Furthermore, Principal Component Analysis (PCA) revealed that samples from the same treatment group clustered closely together in the principal component space and were distinctly separated from other treatment groups (Figure 1B), indicating significant differences in gene expression profiles between treatments.

**Figure 2:**
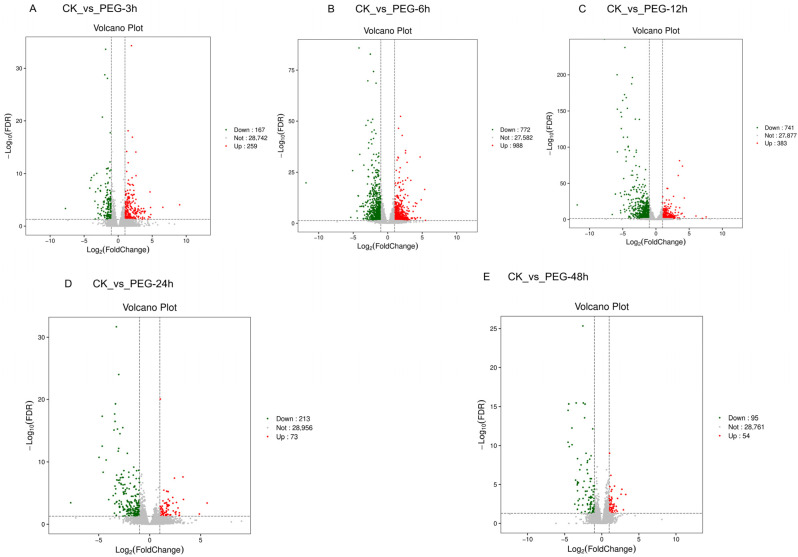
Volcano plots of differentially expressed genes (DEGs) at different time points under PEG-induced osmotic stress. (**A**) Volcano plot of DEGs in CK (0 h) vs. PEG 3 h. (**B**) Volcano plot of DEGs in CK (0 h) vs. PEG 6 h. (**C**) Volcano plot of DEGs in CK (0 h) vs. PEG 12 h. (**D**) Volcano plot of DEGs in CK (0 h) vs. PEG 24 h. (**E**) Volcano plot of DEGs in CK (0 h) vs. PEG 48 h. The vertical dashed lines indicate the threshold of |log2(fold change)| = 1, and the horizontal dashed line represents the significance threshold (FDR = 0.05).

Based on these findings, differential expression analysis was conducted on the transcriptome data across the time course. Compared to the control (0 h), a total of 426, 1760, 1124, 286, and 149 differentially expressed genes (DEGs) were identified in leaf samples collected at 3, 6, 12, 24, and 48 h under PEG-induced osmotic stress, respectively (Figure 2A–E). Notably, the number of DEGs peaked at 6 h, with both upregulated and downregulated genes reaching their maximum levels. Further intersection analysis revealed that the DEGs at different time points exhibited both overlaps and distinct temporal specificity, with 15 genes found to be commonly differentially expressed across all time points (3, 6, 12, 24, and 48 h) (Figure S1A,B).

### 3.2. GO Enrichment and KEGG PathwayAnalyses of Differentially Expressed Genes

**Figure 3:**
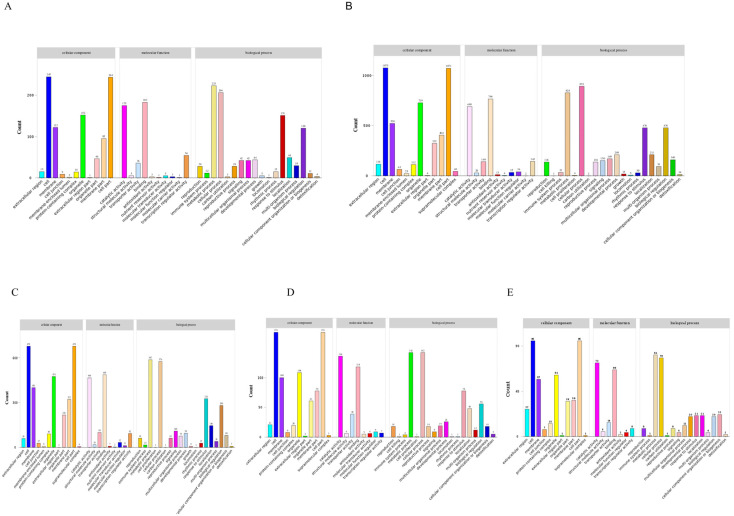
Gene Ontology (GO) enrichment analysis of DEGs at different stages of PEG-induced osmotic stress. The top significantly enriched GO terms are categorized into three main ontologies: Biological Process (BP), Cellular Component (CC), and Molecular Function (MF). Comparisons are presented for (**A**) CK vs. PEG-3h, (**B**) CK vs. PEG-6h, (**C**) CK vs. PEG-12h, (**D**) CK vs. PEG-24h, and (**E**) CK vs. PEG-48h.

Gene Ontology (GO) functional annotation categorizes differentially expressed genes (DEGs) into three major classes: Biological Process (BP), Cellular Component (CC), and Molecular Function (MF). In this study, GO functional annotation was performed on DEGs identified between osmotic stress treatments (3, 6, 12, 24, and 48 h) and the control, followed by statistical analysis of the top-enriched GO terms at each time point. As the duration of stress increased, the number of DEGs exhibited a trend of initially increasing and then decreasing, peaking at 6 h and 12 h, which indicates that the transcriptional response was most active during this phase (Figure 3).

During the early stage of osmotic stress (3 h), enriched GO terms were primarily concentrated in the Biological Process and Molecular Function categories. The DEGs were mainly involved in metabolic process, cellular process, and response to stimulus. At 6 h and 12 h, both the number and functional diversity of enriched GO terms increased significantly, with DEGs distributed across all three GO categories. Within the Biological Process category, metabolic process, cellular process, response to stimulus, and biological regulation were dominant. In the Molecular Function category, binding and catalytic activity predominated, while transporter activity and transcription regulator activity were notably enhanced. Regarding the Cellular Component, DEGs were primarily localized to the cell, organelle, and membrane. As the treatment duration extended to 24 h and 48 h, the number of DEGs and functional categories tended to converge, with enriched GO terms mainly related to metabolic process and cellular process (Table S4).

**Figure 4:**
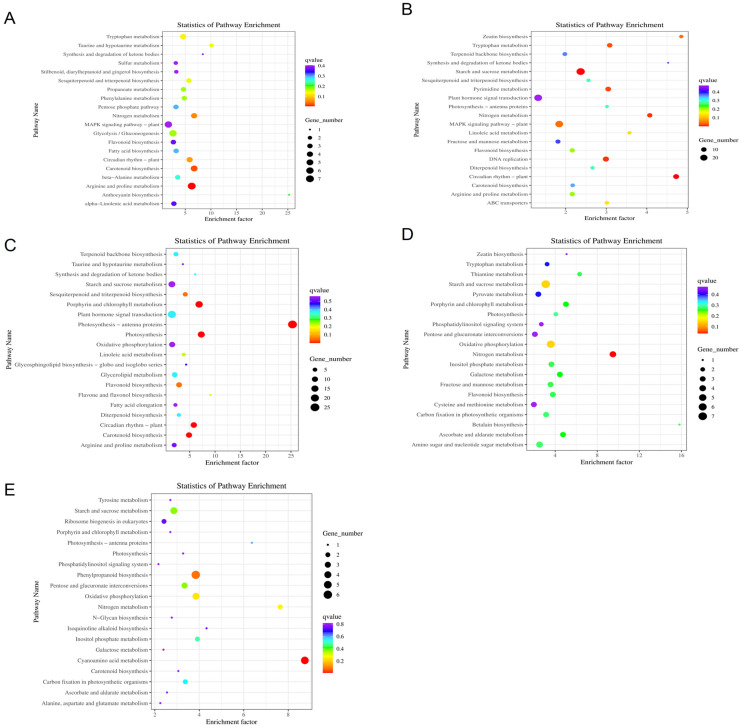
KEGG pathway enrichment analysis of DEGs in Pyrus betulifolia under PEG-induced osmotic stress. (**A**) CK vs. PEG-3h, (**B**) CK vs. PEG-6h, (**C**) CK vs. PEG-12h, (**D**) CK vs. PEG-24h, (**E**) CK vs. PEG-48h. The y-axis shows the names of the KEGG pathways, and the x-axis represents the Rich Factor (the ratio of the number of DEGs to the total number of genes in a given pathway). The color of the dots indicates the q-value (adjusted *p*-value), with redder colors representing higher significance. The size of the dots corresponds to the number of DEGs enriched in each pathway.

KEGG pathway enrichment analysis of DEGs across different osmotic stress treatment times is presented in Figure 4. Pathways such as Nitrogen metabolism, Phenylpropanoid biosynthesis, and Linoleic acid metabolism were enriched at multiple time points. DEGs specifically enriched only during the early stage (3 h) were primarily involved in pathways including the MAPK signaling pathway, Arginine and proline metabolism, and Tyrosine metabolism. During the intermediate stage (6 h and 12 h), genes related to DNA replication, Mismatch repair, Pyrimidine metabolism, and Plant hormone signal transduction showed the most active expression. In the late stages of stress (24 h and 48 h), the focus of enrichment shifted; pathways such as Starch and sucrose metabolism, Porphyrin and chlorophyll metabolism, Ribosome biogenesis in eukaryotes, and Protein processing in endoplasmic reticulum were significantly enriched. This suggests that in the later stages, the plant adapts to persistent water deficit primarily by regulating carbon metabolism, energy supply, and protein homeostasis.

### 3.3. Identification of Key Drought-Responsive Co-Expression Modules and Temporal Pattern Analysis

**Figure 5:**
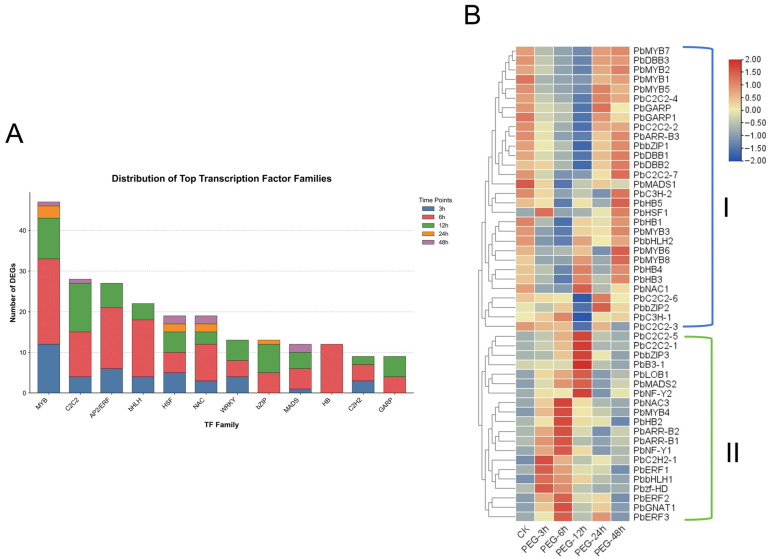
Temporal dynamic response analysis of transcription factor families under PEG-induced osmotic stress. (**A**) Stacked bar chart showing the distribution of the top 12 differentially expressed transcription factor (TF) families across five osmotic stress treatment time points (3 h–48 h). Different colors represent different time points, and bar heights indicate the number of differentially expressed genes (DEGs). The “Others” category and unclassified families were excluded. (**B**) Hierarchical clustering heatmap of the top 50 transcription factors with the highest expression variance. Rows represent individual TF genes (labeled as: Gene Name) (Table S5), and columns represent treatment times.Two major expression patterns were identified: Cluster I shows an early decrease followed by recovery, while Cluster II exhibits early induction with peak expression at intermediate time points and a subsequent decline.

To elucidate the dynamic transcriptional regulatory processes induced by osmotic stress, we first identified differentially expressed transcription factors (DETFs) and systematically analyzed their family composition and temporal expression patterns. As illustrated in Figure 5A, the MYB, C2C2, AP2/ERF, and bHLH families were the predominant transcription factor types across all time points, with most families exhibiting distinct stage-specific response characteristics where the number of DETFs peaked at 6 h and 12 h before gradually declining at 24 h and 48 h. Based on the clustering analysis of the top 50 DETFs with the highest inter-sample variance (Figure 5B), these factors were clearly categorized into two distinct temporal dynamic patterns. Group I transcription factors exhibited a “down-then-up” trend, characterized by high expression levels in the CK group, followed by a continuous decline to a nadir as stress progressed, and a subsequent recovery. Conversely, Group II transcription factors displayed an “up-then-down” pattern, where expression levels steadily increased to a peak with the progression of stress before gradually declining.

### 3.4. Temporal Expression Characteristics of Functionally Related Genes Under PEG-Induced Osmotic Stress

**Figure 6:**
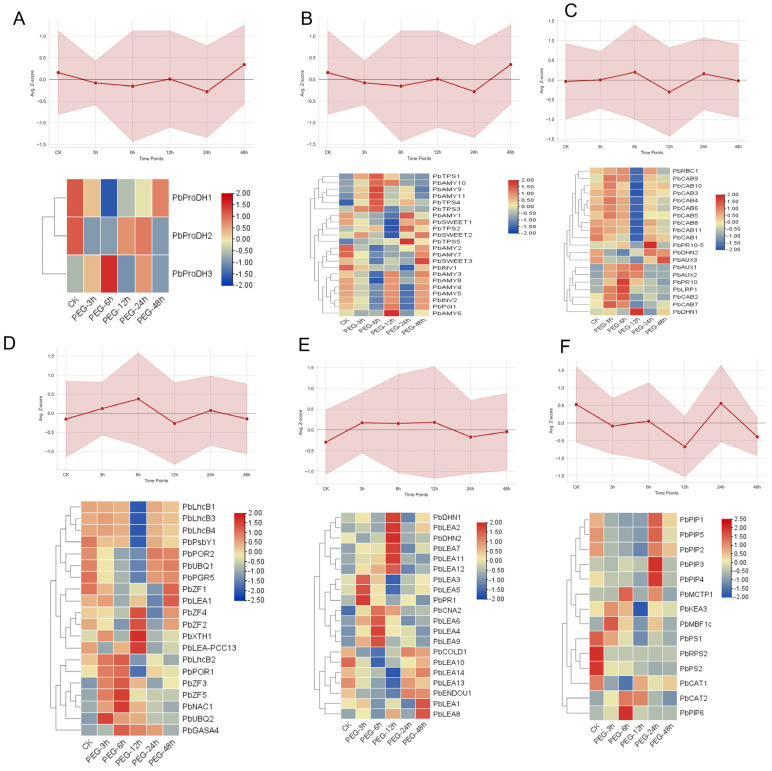
Expression profiles and temporal dynamics of key functional genes involved in the PEG-induced osmotic stress response. (**A**–**F**) Integrated panels showing the expression patterns of representative differentially expressed genes (DEGs) across six functional categories: (**A**) Osmotic adjustment–proline metabolism (Osmotic_Proline), (**B**) Osmotic adjustment–sugar metabolism (Osmotic_Sugar), (**C**) Hormone signaling–ABA pathway (Hormone_ABA), (**D**) Reactive xygen species scavenging system (ROS_Scavenging), (**E**) Protective proteins–LEA (Protection_LEA), (**F**) Water transport (Transport_Water). The upper line plot in each panel illustrates the average expression trend (mean Z-score ± SEM) for the respective category, highlighting the temporal dynamics of each functional group. The lower heatmap displays individual gene expression across the control (CK) and different drought treatment time points (3 h, 6 h, 12 h, 24 h, 48 h). Key genes with the highest expression variance were selected for display.

To elucidate the temporal dynamics of gene expression in *Pyrus betulifolia* under PEG-induced osmotic stress, we analyzed DEGs associated with osmotic adjustment, hormone signaling, ROS scavenging, and cellular protection. As shown in Figure 6, these functional modules exhibited distinct temporal response patterns. regarding osmotic adjustment, Osmotic_Proline genes (Figure 6A) displayed a slight downward trend during the early-to-mid stages, followed by a marked increase at the late stage (48 h). In contrast, Osmotic_Sugar genes (Figure 6B) were significantly downregulated at 6–12 h but exhibited a rapid recovery to high expression levels during the late phase (24–48 h). In terms of hormone signaling, Hormone_ABA genes (Figure 6C) showed rapid upregulation at 3 h and peaked at 6 h; this was followed by a transient decline and a subsequent rebound at 24 h. ROS_Scavenging genes (Figure 6D) were significantly induced primarily during the mid-stress stage, whereas Protection_LEA genes (Figure 6E) were characterized by rapid early upregulation followed by sustained fluctuating expression. Conversely, Transport_Water genes (Figure 6F) showed an overall suppressive trend, with the most significant downregulation occurring at 12 h.

### 3.5. Identification and Temporal Analysis of Key Co-Expression Modules Responding to PEG-Induced Osmotic Stress

**Figure 7:**
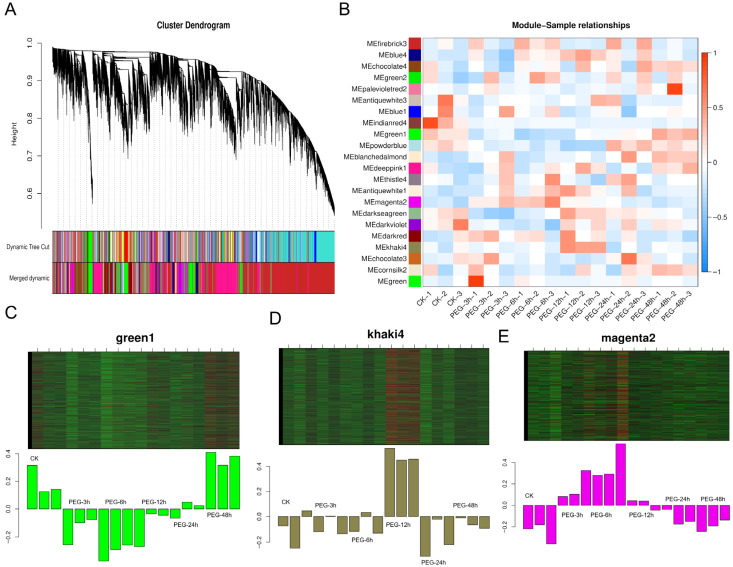
Construction of gene co-expression networks. (**A**) Hierarchical clustering dendrogram of genes showing module assignment generated by WGCNA. (**B**) Heatmap of correlations between modules and sample traits. (**C**) Expression profile of the MEgreen1 module across different time points. (**D**) Expression profile of the MEkhaki4 module. (**E**) Expression profile of the MEmagenta2 module.

**Figure 8:**
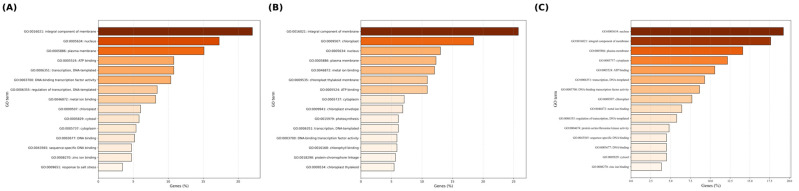
GO enrichment analysis of differentially expressed genes (DEGs) within key WGCNA modules. The top 15 enriched GO terms in (**A**) Green1 module, (**B**) MEkhaki4 module, (**C**) MEmagenta2 module are displayed based on the percentage of genes. The x-axis represents the gene percentage (Genes %), and the y-axis shows the GO terms with their respective IDs. Color intensity indicates the magnitude of gene enrichment.

**Figure 9:**
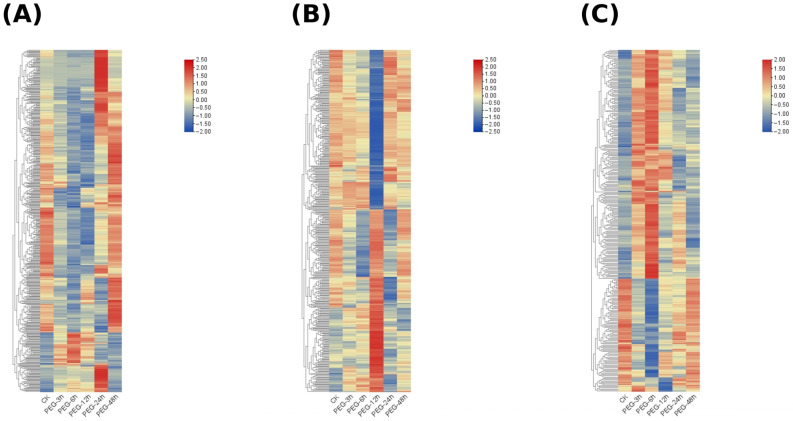
Gene expression heatmaps for the three specific co-expression modules. The hierarchical clustering heatmaps illustrate the dynamic expression patterns of differentially expressed genes (DEGs) within the (**A**) MEgreen1, (**B**) MEkhaki4, (**C**) MEmagenta2 modules under drought stress. Rows represent individual genes, and columns represent different treatment time points (CK, PEG-3h, 6h, 12h, 24h, and 48h). The color scale indicates the relative expression levels after row-based Z-score normalization (Log_2_ based), where red signifies up-regulation and blue signifies down-regulation.

To elucidate the key gene networks regulating drought tolerance in plants, this study utilized Weighted Gene Co-expression Network Analysis (WGCNA) on time-series samples under Identification and Temporal Analysis of Key Co-expression Modules Responding to PEG-Induced Osmotic Stress. Using the dynamic tree cut method, a total of 22 co-expression modules were identified (Figure 7A). Among them, three modules exhibited significant and distinct temporal response characteristics (Figure 7B): the MEkhaki4 module (2811 genes) showed a specific transient induction at 12 h (Figure 7D); the MEmagenta2 module (1504 genes) maintained high expression levels during the mid-stress stage (12–24 h) (Figure 7E); and the MEgreen1 module (2343 genes) displayed a significant late-stage specific response, peaking at 48 h (Figure 7C). In contrast, the MEindianred4 module was significantly suppressed by drought stress. The biological functions of genes within the Green1, MEkhaki4, and MEmagenta2 modules were characterized using Gene Ontology (GO) enrichment analysis (Figure 8). The Green1 module predominantly comprised genes associated with the integral components of the membrane and nucleus. Notably, this module exhibited a marked enrichment in core regulatory categories, specifically DNA-binding transcription factor activity and the regulation of transcription. Such findings underscore its pivotal role in orchestrating transcriptional responses under stress conditions (Figure 8A). In contrast, the MEkhaki4 module was uniquely enriched in terms directly linked to photosynthetic activity—such as chloroplast, photosynthesis, and chlorophyll binding—over and above fundamental membrane components (Figure 8B). Simultaneously, the MEmagenta2 module demonstrated a high degree of correlation with nuclear localization and DNA-templated transcription, alongside essential signal transduction pathways, including protein serine/threonine kinase activity and protein phosphorylation (Figure 8C). Among the three clustering modules, differential genes exhibited distinct dynamic expression patterns (Figure 9). Some genes showed peak expression levels in the control group (CK), followed by a sustained decline with prolonged drought stress (PEG treatment). Conversely, other genes demonstrated gradual up-regulation over time. Additionally, certain genes exhibited a fluctuating trend of initial increase followed by decline, reaching expression peaks during the mid-stress phase (e.g., 6 h or 12 h).

### 3.6. Identification of Core Regulatory Hubs in Drought-Responsive Modules

**Figure 10:**
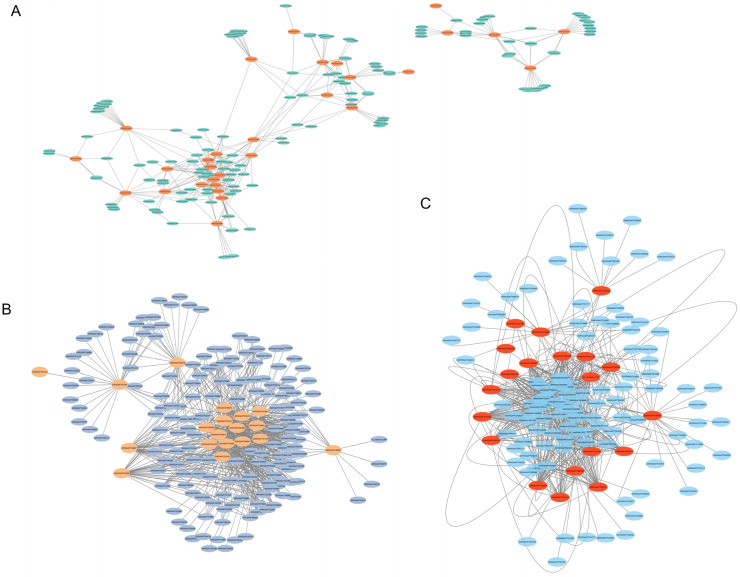
Identification of hub transcription factor genes in the three key co-expression modules. (**A**) Hub transcription factors identified in the MEgreen1 module. (**B**) Hub transcription factors identified in the MEkhaki4 module. (**C**) Hub transcription factors identified in the MEmagenta2 module.

Hub genes were defined as those with the highest intramodular connectivity within each module [17]. In the MEgreen1, MEkhaki4, and MEmagenta2 modules, a large number of differentially expressed genes (DEGs) were annotated as transcription factors, mainly belonging to the MYB, bZIP, and C2H2 families. Based on the hub gene-centered co-expression networks and their expression patterns, six transcription factors with important potential regulatory roles were selected from these three modules for further analysis, including the pseudo-response regulator 5 (PRR5) homolog GWHGAAYT000932, CONSTANS-LIKE 9 (COL9) homolog GWHGAAYT029012, nuclear transcription factor Y subunit C9 (NF-YC9) homolog GWHGAAYT014779, bZIP transcription factor EMBP-1 homolog GWHGAAYT015008, CONSTANS-LIKE 6 (COL6) homolog GWHGAAYT019191, and the MYB transcription factor REVEILLE 1 (RVE1) homolog GWHGAAYT056362 (Figure 10).

### 3.7. qRT-PCR Validation of Differentially Expressed Genes

**Figure 11:**
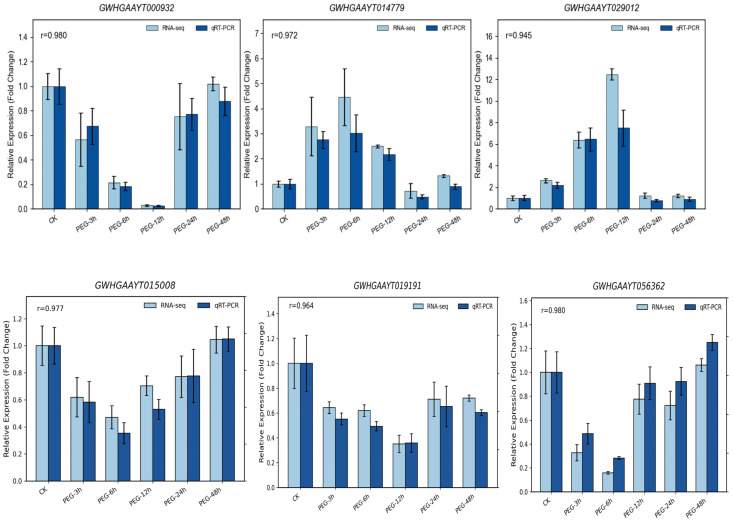
qRT-PCR validation of RNA-seq results for representative drought-responsive genes in Pyrus betulifolia. The relative expression levels of six selected genes were analyzed at different time points (0, 3, 6, 12, 24, and 48 h) under PEG-induced osmotic stress. Gene expression levels were normalized to the reference gene Actin and calculated using the 2^−ΔΔCt^ method. Data represent means ± SD of three biological replicates. Pearson’s correlation coefficients (r) between RNA-seq and qRT-PCR results are shown in each panel.

To validate the reliability of RNA-seq data and confirm the expression patterns of the identified key regulatory hub genes, these six representative core genes were selected for qRT-PCR verification. As shown in Figure 11 and Figure S2, although there were slight fluctuations in absolute fold change values due to inherent sensitivity differences between the two technologies, the expression trends detected by qRT-PCR were highly consistent with the FPKM profiles obtained from RNA-seq.

## 4. Discussion

For perennial woody fruit trees such as the pear, drought tolerance is largely determined by the type of rootstock used. As a widely utilized and superior rootstock, *Pyrus betulifolia* exhibits significant drought resistance potential, making it a critical material for investigating drought adaptation mechanisms [18]. In this study, systematic transcriptomic profiling of leaves across multiple time points under PEG-induced osmotic stress revealed pronounced time-dependent regulatory dynamics during the stress response. The strong reproducibility among biological replicates and the clear transcriptomic separation among treatments underscore the robustness of our experimental design and the reliability of the sequencing data, providing a solid foundation for subsequent identification of differentially expressed genes and regulatory pathways. Notably, the number of differentially expressed genes (DEGs) peaked at 6 h, suggesting that this stage may represent a critical transition from stress perception to extensive transcriptional reprogramming. In contrast, only a limited number of DEGs were detected at 3 h, mainly reflecting the rapid perception of drought signals and the initiation of early stress responses. As the duration of stress extended beyond 12 h, the number of DEGs gradually decreased, with a pronounced decline observed at 24 h and 48 h, indicating an attenuation of transcriptional regulation intensity and suggesting that the plant may enter a relatively stable regulatory phase at the transcriptional level. It is noteworthy that the composition of differentially expressed genes (DEGs) varied markedly across different time points, indicating a clear phase-specific transcriptional response of *Pyrus betulifolia* to drought stress. At the early stage of stress (3 h), only a limited number of genes were rapidly activated, primarily involving pathways related to stress perception, hormone signal transduction, and initial osmotic adjustment, reflecting the rapid sensing and primary response of plants to water deficit. As stress progressed to 6 h and beyond, the number of DEGs increased substantially, with progressive activation of genes associated with carbohydrate metabolism, antioxidant defense, ionic homeostasis, and transcription factor regulation, suggesting a transition from transient stress signaling to extensive transcriptional reprogramming. This temporal regulatory pattern is highly consistent with the drought-responsive characteristics reported in Brassica oleracea [19], particularly in the coordinated regulation of starch and sucrose metabolism, osmolyte biosynthesis, and reactive oxygen species scavenging pathways. Furthermore, a large number of time-specific DEGs were identified between the 6 h and 48 h PEG treatments, which were mainly involved in the regulation of photosynthesis, reprogramming of energy metabolism, and hormone signaling pathways. These results imply that this period represents a critical transitional window during which *P. betulifolia* shifts from short-term stress responses to long-term adaptive regulation. This regulatory feature is in close agreement with the molecular response patterns observed in black cottonwood (*Populus trichocarpa* [20]), including metabolic reprogramming, enhancement of antioxidant defenses, and modulation of photosynthetic processes under drought conditions.

Moreover, functional analysis of PEG-induced DEGs indicated that *P. betulifolia* copes with water deficit primarily by coordinating signal transduction, metabolic regulation, and stress response-related processes. Although specific GO and KEGG enrichment results vary among species, the regulatory patterns revealed in this study are generally consistent with the transcriptional response characteristics of woody plants such as apple [21] under drought stress. Previous studies have shown that under drought conditions, plants typically activate various signaling and metabolic pathways to re-establish cellular ion homeostasis, osmotic balance, and reactive oxygen species (ROS) homeostasis, thereby mitigating stress-induced damage [22]. Functional analysis demonstrated that *P. betulifolia* responds to drought stress mainly by reprogramming metabolic pathways (such as starch and sucrose metabolism, and lipid metabolism) and hormone signaling pathways (such as plant hormone signal transduction). These metabolic adjustments likely facilitate the maintenance of cellular osmotic balance and energy homeostasis, which are fundamental to plant drought resistance [23]. In the early stage of stress (3 h), the MAPK signaling cascade was activated synchronously with proline and arginine metabolism. The significant enrichment of the MAPK signaling pathway further corroborates that the kinase cascade reaction is a core signal transduction mechanism in plant drought response. This pathway typically transmits signals via a MAP3K-MAP2K-MAPK phosphorylation cascade, amplifying the initial drought signals perceived by the cell membrane and transmitting them to the nucleus to activate specific transcription factors, ultimately regulating the expression of drought-related functional genes [24]. Entering the transcriptionally active phase (6–12 h), the response mechanism involved not only extensive hormone signal transduction and transcription factor regulation but also a significant enrichment of DNA repair pathways. This suggests that while mobilizing defense networks, *P. betulifolia* actively initiates genomic protection mechanisms to counter potential genetic damage caused by oxidative stress. As the stress extended to the late stage (24–48 h), the transcriptional focus gradually shifted toward starch and sucrose metabolism and protein processing in the endoplasmic reticulum.

Drought stress induces the expression of a series of transcription factors (TFs) that regulate physiological responses through complex signaling networks to alleviate drought-induced damage and play key roles in plant growth and development. We analyzed co-expression modules highly associated with drought-responsive genes and identified multiple hub transcription factors, including members of the C2H2, DBB, NF-Y, B3, C2C2, WRKY, bZIP, and bHLH families. Previous studies have confirmed that inducing C2H2 overexpression in sorghum significantly improves drought tolerance [25]. In wheat, overexpression of the C2H2 zinc finger transcription factor TaZAT8-5B enhances drought tolerance and root growth in Arabidopsis; its homologous genes regulate the expression of stress-related proteins such as superoxide dismutase and dehydroascorbate reductase, effectively scavenging ROS and thereby enhancing drought resistance [26]. Similar mechanisms have been reported in apple [27] and rice [28]. Additionally, the expression of DBB in wheat [29] and NF-Y in potato [30] has also been proven to increase drought stress tolerance in transgenic plants. Hormone regulation can also enhance drought resistance; for example, the wheat bHLH-type transcription factor gene TabHLH1 enhances cold tolerance by regulating osmotic stress tolerance via ABA-related pathways [31]. Another report indicated that salicylic acid treatment regulates root hair differentiation and growth by upregulating the expression of GLABRA2 (GL2), thereby improving drought tolerance [32].

Through time-series WGCNA, this study revealed a distinct phase-dependent regulatory strategy governing drought adaptation in *P. betulifolia*. Notably, the specific upregulation of chloroplast- and photosynthesis-related genes (MEkhaki4) at the early stage (12 h) suggests a ‘compensatory stability’ mechanism, where the plant prioritizes the transcriptional maintenance of photosynthetic machinery to prevent early oxidative damage [33]. Subsequently, the regulatory focus shifted towards active signal transduction during the mid-stage (12–24 h), as evidenced by the sustained enrichment of protein serine/threonine kinases in the MEmagenta2 module, which likely facilitates the transmission of osmotic signals to the nucleus [34]. Ultimately, the extensive activation of transcription factors in the MEgreen1 module at 48 h marks a critical transition from signal processing to systemic transcriptional reprogramming, establishing a new homeostatic state for long-term survival [35].

In the present study, weighted gene co-expression network analysis (WGCNA) effectively reduced the dimensionality of the transcriptomic data and successfully identified the core modules (MEgreen1, MEkhaki4, and MEmagenta2) responsive to osmotic stress, along with their highly connected internal hub genes. Notably, the genes occupying topological core positions within the network were extensively enriched in classic plant stress-responsive transcription factor families, such as MYB, bZIP, and C2H2 [36,37]. Building upon this network topology, we identified key factors with potential global regulatory roles, including PRR5, COL9, NF-YC9, EMBP-1, COL6, and RVE1, and performed qRT-PCR validation on six representative core node genes (GWHGAAYT000932, GWHGAAYT029012, GWHGAAYT014779, GWHGAAYT056362, GWHGAAYT019191, GWHGAAYT015008). The results demonstrated that although the absolute fold-change values exhibited minor fluctuations due to the inherent sensitivity differences between the two quantification techniques, the expression trends of these core genes were highly consistent with the FPKM profiles derived from RNA-seq. This not only strongly substantiates the high reliability of the current RNA-seq dataset but also further implies that these hub transcription factors act as critical molecular switches in the response of *Pyrus betulifolia* to PEG-induced osmotic stress, collectively driving the hierarchical transcriptional reprogramming of the downstream drought-protective network.

## 5. Conclusions

Using in-depth time-course transcriptome sequence analysis, we found several hypothetical transcription factors (such asWRKY, NAC, and BZIP), whose functions have already been implicated in the regulation of drought stress in other species. We suggest that these genes may have played an important role in the response of pear to drought stress. However, there are still limitations of our research, and although some genes have been identified, there remain deficiencies in testing their function. Future studies should focus on examining the function of these genes.

## Data Availability

The sequence data for expression pattern analysis of PbrLhc genes in response to irrigation or drought conditions is available at NCBI: PRJNA655255. The raw data are available in the Supplementary Materials.

## Supplementary Materials

The following supporting information can be downloaded at: https://www.mdpi.com/article/10.3390/biology15060459/s1, Figure S1: Venn diagram of differentially expressed genes (DEGs) at different time points under PEG-induced drought stress; Figure S2: Correlation analysis of gene expression levels between RNA-seq and qRT-PCR; Table S1. Real-time quantitative PCR primers; Table S2: Sequencing reads and reads mapping of RNA-sequencing; Table S3: Sequencing reads and reads mapping of RNA-sequencing; Table S4: Statistics of GO Enrichment Analysis for Differentially Expressed Genes; Table S5: Correspondence between Gene Names and Gene IDs.
