# Supplementary material for: Deciphering the Temporal Transcriptional Dynamics and Key Regulatory Networks of Pyrus betulifolia in Response to PEG-Induced Osmotic Stress
Source: Biology (Basel). 2026 Mar 11;15(6):459. doi: 10.3390/biology15060459 (PMC13024373; doi:10.3390/biology15060459)
Supplement: Supplementary file 1 [file biology-15-00459-s001.zip › biology-4142893-supplementary.pdf]

Table S1. Real-time quantitative PCR primers.

Name	Forward Primers (5′-3′)	Reverse Primers (5′-3′)
Actin	GCGGTTATGCCCTCCCTC	CGATTTCCCGTTCAGCAGTAG
GWHGAAYT000932	CCAAAGGGAAGCAGCTCTGA	TGGTTCCATCCCGACAAACA
GWHGAAYT029012	TGCGAGCAGGAAATGGGATT	CGCAGCATTCACTTCATCCG
GWHGAAYT014779	AGAGTTGACAATGCGGTCGT	TGATGCAAGCACCTCGTCTT
GWHGAAYT056362	GTGTCTGGCCGAGAGAGATG	GACTAACACAGGAGGACGCC
GWHGAAYT019191	GCAAGCAGACATGAGAGGGT	CTACTTTGAGCCCCCGTGAG
GWHGAAYT015008	GGAATGGACTCCAACCCCAG	CACTGTGTTTGACACTGCCG

Table S2. Sequencing reads and reads mapping of RNA-sequencing.

Sample	ReadSum (n)	BaseSum(n)	GC(%)	Q20(%)	Q30(%)	Mapped reads (n)	Mapped ratio (%)
CK-1	22188452	6700912504	46.81	98.88	96.86	40677051	91.66
CK-2	20880928	6306040256	47.10	98.89	96.87	38494041	92.18
CK-3	25048205	7564557910	46.91	98.88	96.83	46193826	92.21
PEG-3h-1	25142099	7592913898	47.27	98.93	96.95	46262968	92.00
PEG-3h-2	23286061	7032390422	46.89	98.85	96.79	42712533	91.71
PEG-3h-3	25357824	7658062848	46.78	98.93	96.97	46520635	91.73
PEG-6h-1	25202220	7611070440	47.15	98.92	96.94	46390978	92.04
PEG-6h-2	23550962	7112390524	46.92	98.88	96.86	43296250	91.92
PEG-6h-3	24676000	7452152000	47.02	98.87	96.84	45376640	91.94
PEG-12h-1	23759773	7175451446	46.64	98.90	96.91	43681758	91.92
PEG-12h-2	24831131	7499001562	46.49	98.87	96.85	45507983	91.63
PEG-12h-3	26425290	7980437580	46.75	98.91	96.91	48505543	91.78
PEG-24h-1	22954188	6932164776	47.03	98.88	96.85	42270752	92.08
PEG-24h-2	23845705	7201402910	46.78	98.88	96.85	43860377	91.97
PEG-24h-3	23964353	7237234606	47.20	98.86	96.82	44140130	92.10
PEG-48h-1	24419471	7374680242	46.75	98.89	96.88	44876167	91.89
PEG-48h-2	24753469	7475547638	46.66	98.92	96.96	45491434	91.89
PEG-48h-3	23507556	7099281912	46.65	98.88	96.85	43129269	91.73

Table S3. Sequencing reads and reads mapping of RNA-sequencing.

Group	Sample size	Mean correlation coefficient
CK	3	0.9529
PEG-3h	3	0.9573
PEG-6h	3	0.9584
PEG-12h	3	0.9716
PEG-24h	3	0.9526
PEG-48h	3	0.9888

Table S4. Statistics of GO Enrichment Analysis for Differentially Expressed Genes

classfi	Name	NumberofDEGs				
		3h	6h	12h	24h	48h
cellular component	extracellular region	15	116	59	21	27
	cell	245	1075	678	176	95
	membrane	122	520	401	100	57
	cell junction	9	63	27	8	7
	membrane-enclosed lumen	2	23	8	0	0
	protein-containing complex	14	112	89	20	13
	organelle	152	729	474	108	61
	extracellular region part	1	4	1	2	1
	organelle part	46	322	218	61	35
	membrane part	95	404	322	78	36
	supramolecular complex	0	46	6	3	1
molecular function	catalytic activity	175	689	466	136	73
	structural molecule activity	5	29	18	6	5
	transporter activity	36	140	99	39	14
	binding	183	766	485	118	66
	antioxidant activity	3	13	7	5	2
	nutrient reservoir activity	1	4	2	0	0
	molecular transducer activity	5	35	32	6	0
	molecular function regulator	3	39	11	9	4
	molecular carrier activity	1	2	0	0	0
	transcription regulator activity	54	143	92	7	8
biological process	reproduction	28	136	61	18	8
	cell killing	0	1	0	1	0
	immune system process	12	33	15	4	1
	metabolic process	223	828	587	142	81
	cell proliferation	0	4	1	1	0
	cellular process	206	891	574	142	78
	carbon utilization	3	2	1	0	1
	reproductive process	28	136	61	18	8
	signaling	42	150	109	9	4
	multicellular organismal process	42	169	75	19	11
	developmental process	44	209	94	26	20
	growth	5	18	8	0	0
	locomotion	1	6	1	1	0
	rhythmic process	15	30	27	1	0
	response to stimulus	151	476	326	78	21
	localization	49	211	146	48	21
	multi-organism process	29	91	39	12	4
	biological regulation	120	476	280	56	20
	cellular component organization or biogenesis	11	160	80	18	22
	detoxification	4	14	7	5	2

Table S5: Correspondence between Gene Names and Gene IDs

ID	Symbol
GWHGAAYT011454	PbMYB7
GWHGAAYT054569	PbC2C2-5

GWHGAAYT025071	PbNAC3
GWHGAAYT028125	PbHB1
GWHGAAYT051402	PbC2H2-1
GWHGAAYT015617	PbC2C2-6
GWHGAAYT030469	PbGARP
GWHGAAYT038153	PbERF1
GWHGAAYT049817	PbbZIP2
GWHGAAYT054167	PbLOB1
GWHGAAYT013386	PbERF2
GWHGAAYT000887	PbHB4
GWHGAAYT018938	PbC2C2-2
GWHGAAYT009070	PbNAC1
GWHGAAYT050430	PbARR-B2
GWHGAAYT012964	PbMYB4
GWHGAAYT027897	PbMADS2
GWHGAAYT006317	PbbHLH2
GWHGAAYT056034	PbGARP1
GWHGAAYT032278	PbbZIP3
GWHGAAYT021447	PbNF-Y2
GWHGAAYT000955	PbMYB5
GWHGAAYT046922	PbC2C2-3

GWHGAAYT020039	PbC3H-1
GWHGAAYT028559	PbERF3
GWHGAAYT037945	PbDBB2
GWHGAAYT032228	PbDBB3
GWHGAAYT028503	PbC2C2-4
GWHGAAYT049050	PbMYB6
GWHGAAYT032488	PbC2C2-7
GWHGAAYT000932	PbARR-B3
GWHGAAYT055110	PbMYB8
GWHGAAYT031639	PbC3H-2
GWHGAAYT049085	PbHB5
GWHGAAYT056362	PbMYB3
GWHGAAYT052915	PbbHLH1
GWHGAAYT051518	PbbZIP1
GWHGAAYT025098	Pbzf-HD
GWHGAAYT056110	PbDBB1
GWHGAAYT002205	PbARR-B1
GWHGAAYT018872	PbHB3
GWHGAAYT014779	PbNF-Y1
GWHGAAYT052832	PbMADS1
GWHGAAYT050722	PbB3-1

GWHGAAYT029012	PbC2C2-1
GWHGAAYT021489	PbGNAT1
GWHGAAYT039049	PbMYB2
GWHGAAYT009227	PbHB2
GWHGAAYT019979	PbHSF1
GWHGAAYT030861	PbMYB1

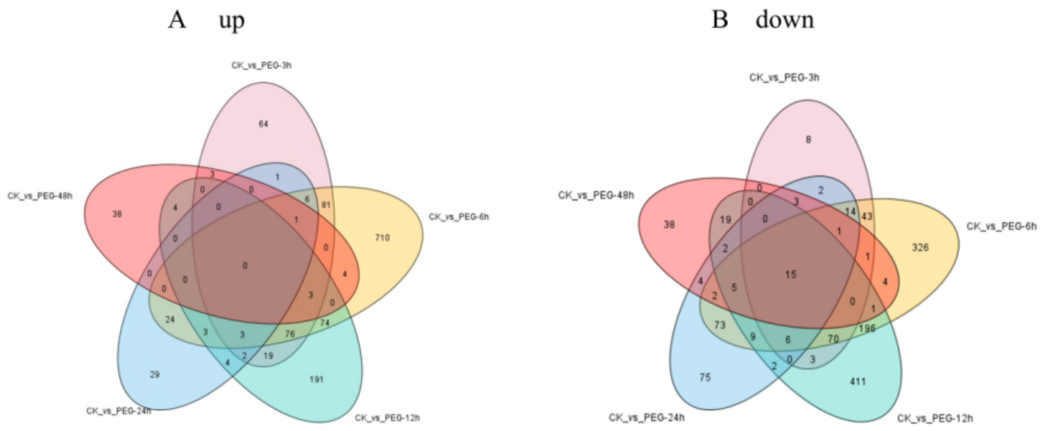

Figure S1. Venn diagram of differentially expressed genes (DEGs) at different time points under PEG-induced drought stress. (A) Venn diagram of up-regulated DEGs, illustrating the unique and shared genes among the five treatment groups (PEG-3h, 6h, 12h, 24h, and 48h) compared to the control (CK). (B) Venn diagram of down-regulated DEGs. The numbers in the overlapping and non-overlapping areas represent the quantity of shared and stage-specific DEGs, respectively.

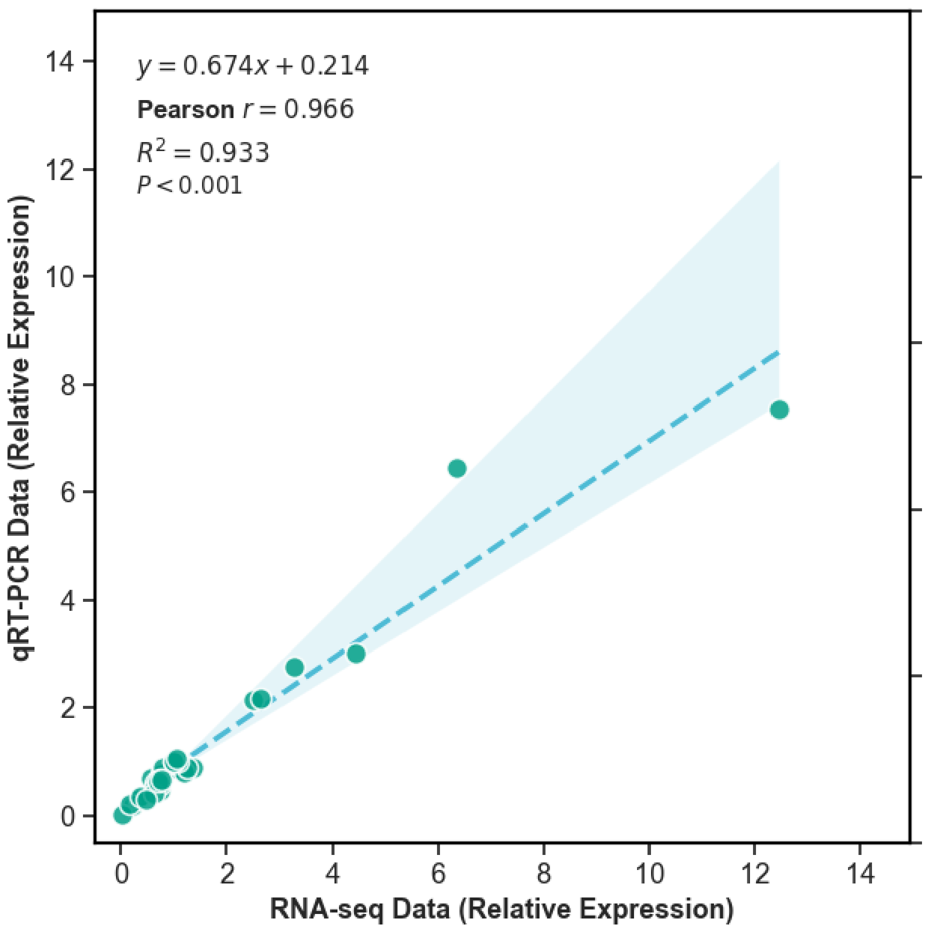

Figure S2. Correlation analysis of gene expression levels between RNA-seq and qRT-PCR. The scatter plot represents data points from 6 representative genes across 6 time points (n=18). The x-axis and y-axis show the relative expression levels (or Log2 Fold Change) obtained from RNA-seq and qRT-PCR, respectively. The black dashed line indicates the linear regression fit, and the gray shaded region represents the 95% confidence interval. The Pearson correlation coefficient (r=0.966) and statistical significance(P<0.0001) indicate a high concordance between the two methods.
